# Complete genome sequence of Jobypre, an L3 subcluster mycobacteriophage isolated from marsh soil in Charleston, South Carolina

**DOI:** 10.1128/mra.00858-25

**Published:** 2025-10-29

**Authors:** Riley M. Polic, Christa P. Joby, James C. Herring, Patrick L. Arnsberger, Ashanti M. Carter, Charlotte E. Drainville, Della R. Evans, Alyson J. Fincke, Calvin G. Geisel, Terranee E. Hines, Ryochi A. Jimenez, Joshua J. King, Malori K. Lesesne, Sophia G. Morrison, Emma P. Peluso, Vineel Prathipati, Cara M. Samson, Mattie C. Tharp, Mouna S. DiBenedetto, Christine A. Byrum

**Affiliations:** 1 Department of Biology, College of Charleston 2343, Charleston, South Carolina, USA; Queens College Department of Biology, Queens, New York, USA

**Keywords:** mycobacteriophage, annotation, *Vilmaviridae*, SEA-PHAGES, Jobypre, genome, actinobacteriophage, bacteriophage, virus, subcluster L3

## Abstract

Jobypre, an L3 subcluster mycobacteriophage (Family: *Vilmaviridae*) extracted from marsh soil at Joe Riley Waterfront Park in Charleston, South Carolina, has siphovirus morphology and infects *Mycobacterium smegmatis* mc^2^155. The Jobypre genome is 75,624 bp long and contains 128 protein-coding genes, 10 transfer RNA sequences, and no transfer-messenger RNA.

## ANNOUNCEMENT

*Mycobacterium smegmatis*, the bacterial host used here, is a harmless soil bacterium utilized to model pathogens like *Mycobacterium tuberculosis* and *M. abscessus*. To better understand phage biology/evolution and aid investigators developing phage therapies against these pathogens (1, 2), a broad effort (3) was undertaken to isolate and sequence actinobacteriophages, including Jobypre.

**Fig 1:**
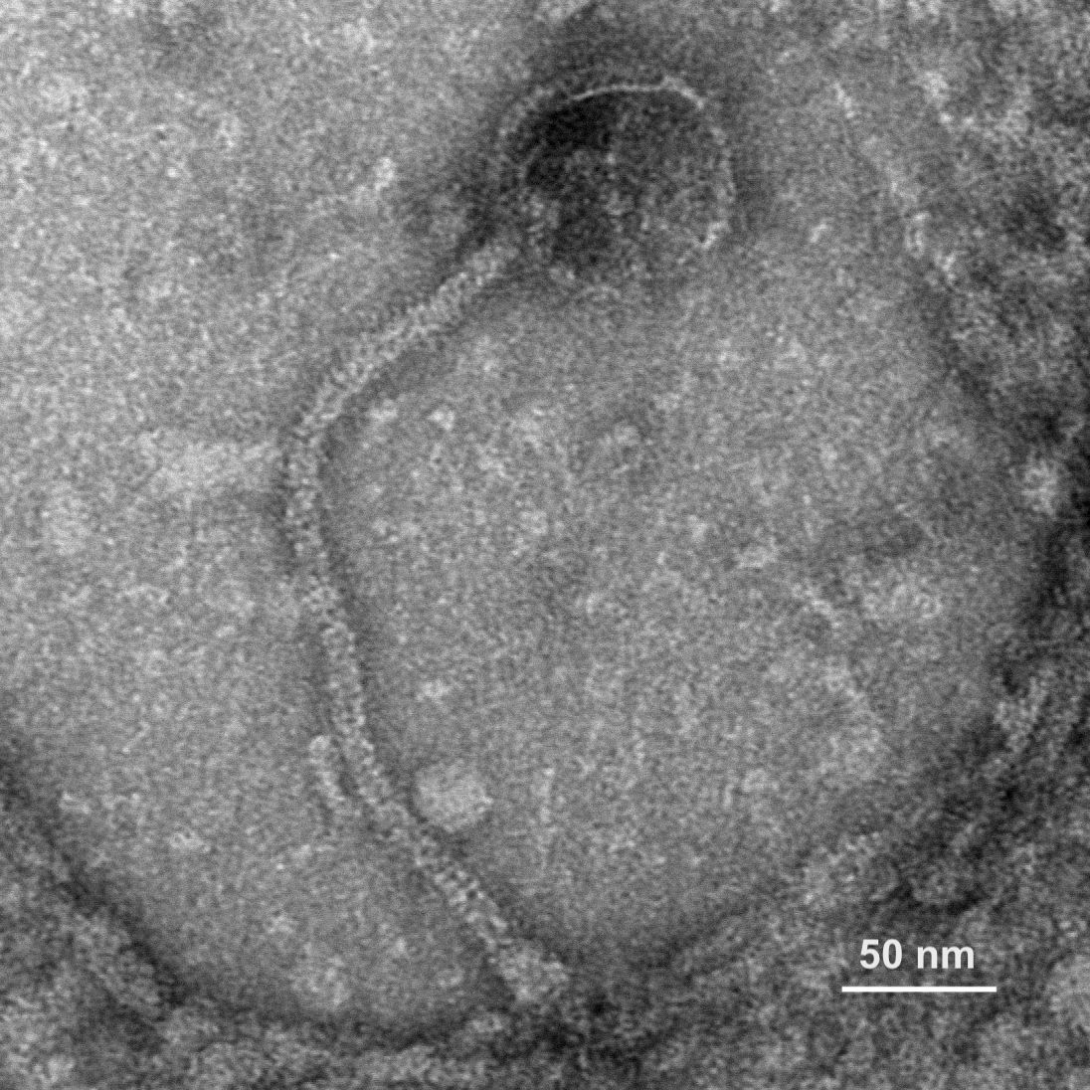
Cluster L3 mycobacteriophage Jobypre exhibits siphovirus morphology. High-titer lysates on Formvar-coated copper grids were negatively stained with 1% uranyl acetate and examined using a JEOL 1010 transmission electron microscope (80 kV) (direct magnification 200,000×; scale bar = 50 nm).

Jobypre was collected from moist surface soil near a palm tree in a marshy area of Joe Riley Waterfront Park in Charleston, South Carolina (32.786463N, 79.938603W). To isolate this virus, soil was added to the 15 mL mark (~14 g) in a 50 mL conical tube and filled to 35 mL with 7H9 broth containing 1 mM CaCl_2_. After agitating 1–2 h (250 rpm, 37°C) and centrifuging (2,000 × *g*, 15 min), the supernatant was filtered (Millipore Steriflip vacuum filters, 0.22 µm pore). This sample was then enriched with *Mycobacterium smegmatis* mc^2^155 at the exponential phase (0.5 mL host culture/25 mL filtrate), shaken/incubated for 72 h (250 rpm, 37°C), and filtered, and the serially diluted filtrates were then plated using the double-layer agar method onto 7H9 agar inoculated with *M. smegmatis* (37°C, 1–3 days) (SEA-PHAGES Discovery Guide contains details) (4). Jobypre forms plaques averaging 0.47 mm in diameter (*n* = 20) (plaque photo at https://phagesdb.org/phages/Jobypre/), and electron microscopy revealed siphovirus morphology with an icosahedral capsid (x̄ = 77.1 nm wide; *n* = 10) and a flexible noncontractile tail (x̄ = 317.8 nm long, 11.6 nm wide; *n* = 10) (Fig. 1).

Phages were purified/amplified over three successive rounds of single-plaque isolation; webbed plates were flooded with phage buffer (incubated 2 h, room temperature); and lysate was collected. DNA from high-titer lysate (4.3 × 10^9^ plaque-forming units/mL) was then extracted for genome analysis (Promega Wizard DNA Cleanup System, Protocol 9.1, Citation 4), and a DNA library was prepared per manufacturer instructions (NEB Ultra II Library Kit). The genome sequencing performed on an Illumina MiSeq System using the Illumina MiSeq Reagent Kit v3 (5) produced 66,557 raw reads (150 bp single-end reads, 57× coverage), which were assembled into a single contig using Newbler v2.9 (6) and checked for accuracy and genome termini on Consed v29.0 (7).

Genome annotation was performed using the workflow tool PECAAN (8), and files were subsequently transferred to DNA Master v5.23.2 (https://phagesdb.org/DNAMaster). Putative genes were identified by consensus with Glimmer v3.02 (9), Starterator v1.1 (10), Genemark v3.25 (11), Phamerator Actino_prophage v606 (12), ARAGORN v1.2.38 (13), and tRNAscan-SE v3.0 (14). Gene functions and domains were predicted by consensus using BLASTp v2.8.2+ (15), HHpred v2.1 (16), TMHMM Deep v1.0.42 (17), SOSUI v1.11 (18), and the NCBI Conserved Domain Database (CDD) (19). Default settings were applied to all programs, except as stated in https://seaphages.org/forums/topic/5398.

The Jobypre genome is 75,624 bp with 59.3% guanine and cytosine (GC) content and contains 128 protein-coding sequences (48 with identified putative functions; two orphan genes, gp67 and gp118; 68 hypothetical proteins with homologs), 10 tRNA sequences, and no tmRNAs (Table 1). This phage is in the L cluster/L3 subcluster (cluster members share >50% nucleotide sequence identity; >35% gene content [GCS] similarity) (20, 21), and the bioinformatic analysis revealed 3′ sticky overhangs at the termini (5′-TCGATCAGCC-3′). The presence of an immunity repressor (gp38 is homologous to proteins preventing phages with same immunity type from infecting the host), tyrosine integrase (gp36), and excise (gp40), as well as turbid plaques, suggest a temperate lifestyle. Whole-genome BLASTn comparison reveals that genomes among members of this subcluster (Family: *Vilmaviridae*) are highly conserved, and that three L3 viruses, namely, Snenia (GenBank KT281794.1), MsGreen (GenBank MK878900.1), and Lumos (GenBank KT372003.1), are nearly identical to Jobypre (>99.9% identity, 100% coverage). Interestingly, of the 24 L3 subcluster bacteriophages currently in the Actinobacteriophage Database (https://phagesdb.org/) (July 2025), all come from tropical or subtropical zones (southeastern US, Texas, South Africa, and Taiwan), except Whirlwind (Pennsylvania; GenBank KF024725.1).

**TABLE 1:** Characteristics of the Jobypre bacteriophage

Parameter	Jobypre data
GenBank accession no.	PV251868.1
SRA accession no.	SRX27257324
Isolation site	Charleston, South Carolina, USA
Collection site coordinates	32.786463 N, 79.938603 W
Isolation host	*Mycobacterium smegmatis* mc^2^155
Genome size (bp)	75,624
Approximate shotgun coverage (x)	57
GC content (%)	59.3
No. of predicted protein-coding genes	128
No. of tRNAs	10
No. of tmRNAs	0
Morphotype	Siphovirus morphology
Cluster	L3
Predicted protein-coding genes (phams) unique to and conserved in all L3 subcluster members:^*a*^ 32,35,46,58,104,106.	

^*a*^ Based on data available in Phamerator on 22 July 2025 (12).

## Data Availability

Jobypre is available at the Pittsburgh Bacteriophage Institute in freezer box 163/grid H8. GenBank and SRA accession numbers appear in Table 1.

## References

[1] Diacon AH, Guerrero-Bustamante CA, Rosenkranz B, Rubio Pomar FJ, Vanker N, Hatfull GF. 2022. Mycobacteriophages to treat tuberculosis: dream or delusion? Respiration 101:1–15. doi:10.1159/000519870 34814151

[2] Nick JA, Dedrick RM, Gray AL, Vladar EK, Smith BE, Freeman KG, Malcolm KC, Epperson LE, Hasan NA, Hendrix J, et al.. 2022. Host and pathogen response to bacteriophage engineered against Mycobacterium abscessus lung infection. Cell 185:1860–1874. doi:10.1016/j.cell.2022.04.024 35568033 PMC9840467

[3] Jordan TC, Burnett SH, Carson S, Caruso SM, Clase K, DeJong RJ, Dennehy JJ, Denver DR, Dunbar D, Elgin SCR, et al.. 2014. A broadly implementable research course in phage discovery and genomics for first-year undergraduate students. mBio 5:e01051-13. doi:10.1128/mBio.01051-13 24496795 PMC3950523

[4] Poxleitner M, Pope W, Jacobs-Sera D, Sivanathan V, Hatfull G. 2018. Phage discovery guide. Howard Hughes Medical Institute, Chevy Chase, MD. Available from: https://seaphagesphagediscoveryguide.helpdocsonline.com/home

[5] Russell DA. 2018. Sequencing, assembling, and finishing complete bacteriophage genomes. Methods Mol Biol 1681:109–125. doi:10.1007/978-1-4939-7343-9_9 29134591

[6] Margulies M, Egholm M, Altman WE, Attiya S, Bader JS, Bemben LA, Berka J, Braverman MS, Chen Y-J, Chen Z, et al.. 2005. Genome sequencing in microfabricated high-density picolitre reactors. Nature 437:376–380. doi:10.1038/nature03959 16056220 PMC1464427

[7] Gordon D, Green P. 2013. Consed: a graphical editor for next-generation sequencing. Bioinformatics 29:2936–2937. doi:10.1093/bioinformatics/btt515 23995391 PMC3810858

[8] Rinehart CA, Gaffney BL, Smith JR, Wood JD. 2016. PECAAN: phage evidence collection and annotation network user guide. Western Kentucky University Bioinformatics and Information Science Center. https://seaphages.org/media/docs/PECAAN_User_Guide_Dec7_2016.pdf.

[9] Delcher AL, Harmon D, Kasif S, White O, Salzberg SL. 1999. Improved microbial gene identification with GLIMMER. Nucleic Acids Res 27:4636–4641. doi:10.1093/nar/27.23.4636 10556321 PMC148753

[10] Pacey M. 2016. Edited by W. Pope. Starterator guide. University of Pittsburgh, Pittsburgh, PA. Available from: https://seaphages.org/media/docs/Starterator_Guide_2016.pdf

[11] Lukashin AV, Borodovsky M. 1998. GeneMark.hmm: new solutions for gene finding. Nucleic Acids Res 26:1107–1115. doi:10.1093/nar/26.4.1107 9461475 PMC147337

[12] Cresawn SG, Bogel M, Day N, Jacobs-Sera D, Hendrix RW, Hatfull GF. 2011. Phamerator: a bioinformatic tool for comparative bacteriophage genomics. BMC Bioinformatics 12:395. doi:10.1186/1471-2105-12-395 21991981 PMC3233612

[13] Laslett D, Canback B. 2004. ARAGORN, a program to detect tRNA genes and tmRNA genes in nucleotide sequences. Nucleic Acids Res 32:11–16. doi:10.1093/nar/gkh152 14704338 PMC373265

[14] Lowe TM, Eddy SR. 1997. tRNAscan-SE: a program for improved detection of transfer RNA genes in genomic sequence. Nucleic Acids Res 25:955–964. doi:10.1093/nar/25.5.955 9023104 PMC146525

[15] Altschul SF, Gish W, Miller W, Myers EW, Lipman DJ. 1990. Basic local alignment search tool. J Mol Biol 215:403–410. doi:10.1016/S0022-2836(05)80360-2 2231712

[16] Söding J, Biegert A, Lupas AN. 2005. The HHpred interactive server for protein homology detection and structure prediction. Nucleic Acids Res 33:W244–8. doi:10.1093/nar/gki408 15980461 PMC1160169

[17] Hallgren J, Tsirigos KD, Pedersen MD, Almagro Armenteros JJ, Marcatili P, Nielsen H, Krogh A, Winther O. 2022. DeepTMHMM predicts alpha and beta transmembrane proteins using deep neural networks. bioRxiv. doi:10.1101/2022.04.08.487609

[18] Hirokawa T, Boon-Chieng S, Mitaku S. 1998. SOSUI: classification and secondary structure prediction system for membrane proteins. Bioinformatics 14:378–379. doi:10.1093/bioinformatics/14.4.378 9632836

[19] Marchler-Bauer A, Derbyshire MK, Gonzales NR, Lu S, Chitsaz F, Geer LY, Geer RC, He J, Gwadz M, Hurwitz DI, Lanczycki CJ, Lu F, Marchler GH, Song JS, Thanki N, Wang Z, Yamashita RA, Zhang D, Zheng C, Bryant SH. 2015. CDD: NCBI’s conserved domain database. Nucleic Acids Res 43:D222–6. doi:10.1093/nar/gku1221 25414356 PMC4383992

[20] Hatfull GF, Jacobs-Sera D, Lawrence JG, Pope WH, Russell DA, Ko C-C, Weber RJ, Patel MC, Germane KL, Edgar RH, et al.. 2010. Comparative genomic analysis of 60 mycobacteriophage genomes: genome clustering, gene acquisition, and gene size. J Mol Biol 397:119–143. doi:10.1016/j.jmb.2010.01.011 20064525 PMC2830324

[21] Hatfull GF. 2020. Actinobacteriophages: genomics, dynamics, and applications. Annu Rev Virol 7:37–61. doi:10.1146/annurev-virology-122019-070009 32991269 PMC8010332

